# The Impact of Insurance and a Usual Source of Care on Emergency Department Use in the United States

**DOI:** 10.1155/2014/842847

**Published:** 2014-02-09

**Authors:** Winston Liaw, Stephen Petterson, David L. Rabin, Andrew Bazemore

**Affiliations:** ^1^Department of Family Medicine, Virginia Commonwealth University, 3650 Joseph Siewick Drive, No. 400 Fairfax, Richmond, VA 22033, USA; ^2^The Robert Graham Center, 1133 Connecticut Avenue, NW Suite 1100, Washington, DC 20036, USA; ^3^Department of Family Medicine, Georgetown University, 4000 Reservoir Road, NW, Washington, DC 20007, USA

## Abstract

*Background*. Finding a usual source of care (USC) is difficult for certain populations. This analysis determines how insurance type and having a USC affect the settings in which patients seek care. *Methods*. In this cross-sectional study of the 2000–2011 Medical Expenditure Panel Surveys, we assessed the percentage of low-income persons with half or more of their ambulatory visits to the emergency department (ED). Respondents were stratified based on insurance type and presence of a USC. *Results*. In 2011, among Medicaid enrollees without USCs, 21.6% had half or more of their ambulatory visits to EDs compared to 8.1% for those with USCs. Among the uninsured without USCs, 24.1% went to an ED for half or more of their ambulatory visits compared to 8.8% for those with USCs in 2011. Among the privately insured without USCs, 7.8% went to an ED for half or more of their ambulatory visits compared to 5.0% for those with USCs in 2011. These differences remained in multivariate analyses. *Conclusions*. Those who lack USCs, particularly the uninsured and Medicaid enrollees, are more likely to rely on EDs.

## 1. Introduction

Between 1996 and 2010, the number of emergency department (ED) visits in the United States (US) increased by 44% [1, 2]. While some patients seek ED care because of convenience, many use emergency services due to lack of access to nonemergency providers [3]. Access to timely, ambulatory care services outside the ED depends on numerous factors, including insurance coverage and having a usual source of care (USC) [4]. As fewer physicians accept Medicaid, it is increasingly difficult for some enrollees to find a USC, leading to an increased number of nonurgent ED visits [5, 6]. Simultaneously, Medicaid expansion as outlined in the Affordable Care Act (ACA) is driving fears that access for the publicly insured will suffer.

Patients treated in the ED for nonurgent issues are often unable to receive primary care outside the ED [7, 8]. Consequently, addressing those barriers can facilitate timely treatment and improve overall allocation of scarce emergency resources. While the relationship between patients with nonurgent complaints and crowding is unclear, increased ED volume has been associated with increased mortality, delays in treatment, and increased rates of patient elopement [9]. Moreover, rising uninsured utilization may contribute to the increasing reluctance of specialists to provide on-call emergency coverage [10–12]. Policymakers need to understand the relationship among insurance coverage, emergency care, and non-ED ambulatory care services in order to appreciate which barriers exist and which populations are affected.

We agree with prior research demonstrating that those with USCs are more likely to visit EDs compared to those without USCs [13] but contend that the existing data do not account for baseline health. Many point to this research as evidence that having a USC does not decrease ED use without acknowledging that those with USCs also have poorer health and are more likely to visit all ambulatory care settings [14]. This analysis adds to the existing literature by adjusting for baseline health in two ways. First, we limit our sample to those with at least one ambulatory care visit. Because we are interested in where patients are receiving care, we exclude patients who do not seek care—typically patients with excellent health and who lack USCs [15]. Second, we use a different outcome measure—whether a person had half or more of their ambulatory care visits to an ED (i.e., the number of ED visits divided by the total number of ambulatory care visits is equal to or greater than 0.50). Studies have documented that having a USC is associated with an increased likelihood of having 1 or more ED visits during a year without controlling for baseline health [13]. To determine whether having a USC affects the setting in which care is received for a patient in poor health, calculating the likelihood of an ED visit is less revealing because their poor health increases the odds that they will seek care. Instead, our approach provides a more valid assessment of whether having a USC affects the setting in which care is received.

Prior research has demonstrated that having a USC decreases the percentage of ambulatory care visits to EDs compared to those without a USC [16]. In this trend analysis, we assess the impact of insurance type and having a USC on where patients receive care. The specific objectives of this analysis are to

1. determine whether having a USC decreases the percentage of persons relying mainly on EDs for ambulatory care among the uninsured, privately insured, and those with Medicaid,
2. determine whether that percentage has changed over time for each insurance group.

## 2. Methods

We conducted a trend analysis of factors influencing the settings in which patients seek care from 2000 to 2011. For each year we used cross-sectional data from the Medical Expenditure Panel Survey (MEPS), a US survey that estimates health services use, medical expenditures, and sources of payment, including insurance coverage. This anonymous, publicly available data set is weighted, allowing estimation of national results. Institutional review board approval was neither required nor obtained.

Because of our interest in care seeking patterns among uninsured and Medicaid patients and desire to limit income's confounding effect, we only included respondents with household incomes less than 200% of the federal poverty level (FPL). Because Medicare ensures near universal coverage for most elderly adults in the US, we further restricted our analysis to persons aged 18–64. Furthermore, we were interested in the health behaviors of respondents using health care services; therefore, we only included those with at least one ambulatory care visit.

Our outcome measure is a dichotomous measure equal to one if half or more of a person's ambulatory care was obtained from an ED. In the MEPS, ambulatory care visits are self- or proxy-reported visits to an outpatient, office-based, or ED setting (which included ED visits resulting in hospitalizations). Our independent variables were insurance status and USC. Health insurance status was classified into three categories: privately insured, Medicaid, and uninsured. Medicaid is a US insurance program for persons of all ages aimed to provide health insurance to those with insufficient resources and is jointly funded by states and the federal government. Individuals were considered privately insured if at any point in a year they were covered by private insurance. Persons were classified as covered by Medicaid if at some point in a year they had Medicaid but never private insurance. The uninsured were individuals that at no point in a year were covered by private or public insurance. USC was ascertained by the question: “Is there a particular doctor's office, clinic, health center, or other place that you usually go to if you are sick or need advice about your health?” Respondents can indicate that the ED is their USC, but the percentage of people in this cohort is small (0.46%). In multivariate models, we included controls for gender, region, age, and self-reported health.

For the baseline year (2000) and final year (2011), we reported the mean number of ED visits, mean number of ambulatory care visits, the mean percentage of ambulatory visits to an ED, and the percentage of persons with half or more of their visits to an ED. The results are stratified by insurance type and USC. In order to determine changes in our outcome measure, for each USC-insurance combination, we calculated year estimates and logistic regression models adjusting for age, race/ethnicity, gender, region, and self-reported health. We opted to use a dichotomous measure instead of the continuous measure capturing the proportion of visits to EDs because of the skewness of this latter measure, with a large number of zeroes and ones. In addition to logistic regression models, we estimated ordinary least squares regression models and multinomial logistic regression models. For all subgroups, we estimated models treating survey year as a continuous measure and a series of dichotomous year indicators. Using coefficients from these models, we obtained adjusted rates for the percentage relying on EDs. While separate models were estimates by insurance status, we used means from the sample as a whole to calculate adjusted rates. With either approach, the results were similar; thus we report the results from the logistic models. When comparing differences between groups, we used the independent sample *t*-test for continuous variables. All analyses were done using STATA 13.1.

## 3. Results

In the 2000–2011 MEPS data, there were 231,683 18–64-year-old respondents. Restricting this sample to persons with income below 200% FPL excluded 145,953 respondents. Additional 32,425 respondents were excluded because they had no ambulatory care visits during the year and further 4,680 were excluded because they were covered by Medicare. After these restrictions, our sample size was 48,653. Excluding cases with missing values for the covariates, 47,565 survey respondents from 2000 to 2011 remained. 14,855 patients were enrolled in Medicaid while 13,477 patients had private insurance. 19,233 patients in this sample were uninsured. The sample sizes ranged from 2,462 in 2000 to 4,546 in 2011.

Regardless of insurance type, patients with USCs report *more* ambulatory care visits compared to patients without USCs, and in 2000, for patients with Medicaid, those with USCs had more visits to EDs compared to those without USCs (Table 1). However, having a USC is associated with a smaller percentage of ambulatory care visits to an ED and a lower likelihood of having most ambulatory care visits to an ED.

The results from the logistic regression analyses reveal other factors associated with a greater reliance on EDs. Differences between racial groups explained some of the effect. Compared to non-Hispanic White Medicaid patients, Black Medicaid patients are more likely to rely mainly on EDs (OR = 1.44 (95% CI 1.24–1.68), *P* < 0.01). We obtained a similar estimate of this race difference for the subset of uninsured and privately insured adults. Women, older persons, and non-Southerners are generally less likely to have half or more of their ambulatory care visits to EDs, across all types of insurance statuses.

**Figure 1:**
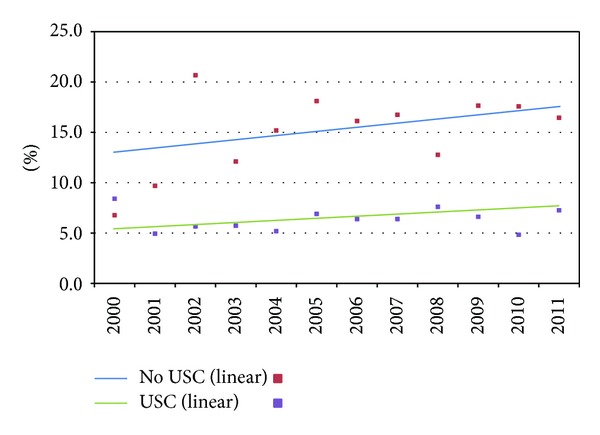
Percentage with half or more of ambulatory visits to EDs for Medicaid enrollees by usual source of care (USC) (2000–2011), adjusted.

In 2011, after adjusting for the aforementioned variables in addition to ethnicity and region of the country, 17.6% of Medicaid beneficiaries without USCs had half or more of their ambulatory care visits to EDs compared to only 7.8% among those with USCs (Figure 1). Between 2000 and 2011, the percentage of Medicaid beneficiaries without USCs relying mainly on an ED increased from an estimated 13.0% to 17.6%. Because of the relatively small number of Medicaid patients without a USC, this increase is not quite significant (*P* = 0.06).

**Figure 2:**
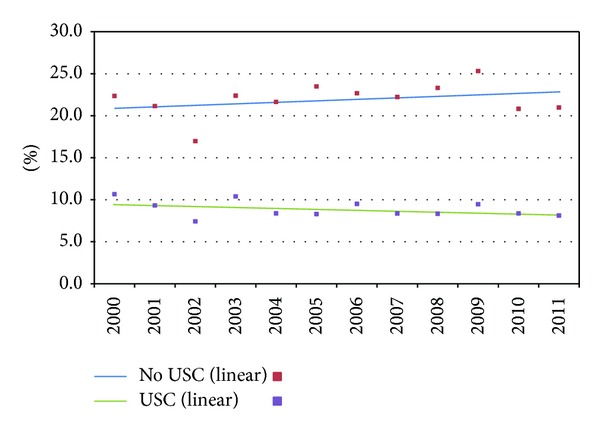
Percentage with half or more of ambulatory visits to EDs for the uninsured by usual source of care (USC) (2000–2011), adjusted.

**Figure 3:**
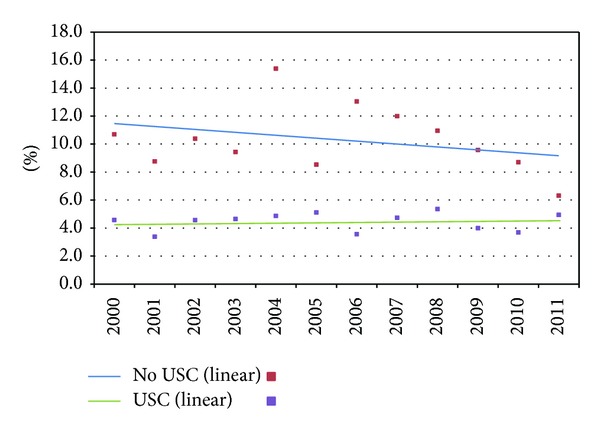
Percentage with half or more of ambulatory visits to EDs for the privately insured by usual source of care (USC) (2000–2011), adjusted.

The difference in reliance on ED between those with and without a USC is greatest among the uninsured (Figure 2). After adjustments, in 2011, 22.4% of the uninsured without a USC had half or more of their visits to an ED, compared to 8.2% for those with a USC. In 2011, 9.3% of the privately insured without a USC had half or more of their visits to an ED, compared to 4.5% for those with a USC (Figure 3). The percentage of the uninsured and privately insured without USCs relying mainly on EDs did not significantly change between 2000 and 2011.

## 4. Discussion

Our results indicate that, for all insurance groups, lacking a USC is associated with a greater reliance on EDs but that the percentage lacking a USC and relying on EDs has not increased significantly over time. Moreover, the utility of having a USC differs for Medicaid patients compared to the privately insured. Although the percentage with a USC is comparable for the privately insured and those with Medicaid [17], Medicaid enrollees are more likely to seek care from EDs. We hypothesize that this discrepancy results from inadequate primary care access to their USC.

Others have found that patients without a USC are less likely to make healthcare visits. Before excluding persons without a visit, we were able to replicate this finding but note that those without USCs are also less likely to seek any care as many are healthy. We limited the analysis to patients who have at least one ambulatory care visit since only patients using health care services decide which sites are accessed. Furthermore, when we restricted the sample to patients with at least one ambulatory care visit, we found that Medicaid patients lacking USCs were more likely to rely on EDs compared to those with USCs.

In light of insurance expansion, policymakers can learn from the events unfolding in Massachusetts where the newly insured initially experienced difficulty accessing USCs. In 2006, reform in Massachusetts dramatically decreased the uninsured through an individual mandate, Medicaid expansion, availability of publicly subsidized insurance obtained through a health insurance connector, and a provision requiring employers to contribute to employee premiums. Following implementation, studies indicated ED utilization did not decrease, causing some to question the legislation's effectiveness [18]. In 2008, Massachusetts ED users reported barriers when trying to access the health care system. Those of low income were more likely to report barriers as ED users were more likely than nonusers to be on public or nonemployer sponsored insurance and have been told that providers were not accepting their insurance or new patients [19]. Concurrently, primary care physician (PCP) panels are full statewide. While 70% of Massachusetts family physicians were accepting new patients in 2007, that figure decreased to 60% in 2008. Of those accepting new patients, the average wait time was 44 days, which increased from 34 days in 2007. The percentage of internists accepting new patients has similarly decreased [20]. The Massachusetts experience shows that reform can decrease the uninsured, but our analysis suggests that those of low income particularly if on Medicaid may have difficulty accessing non-ED ambulatory care as they become newly insured under reform [21]. Furthermore, the trend towards increasing use of EDs by Medicaid patients, who will account for some 11 to 16 million of the 40 million newly insured, suggests ED demand will continue to be a problem [22].

We hypothesize that increasing access to primary care may free up scarce emergency resources though our analysis did not specifically address this question. Unfortunately, fewer US medical graduates are entering primary care [23]. Addressing the supply of PCPs and provider maldistribution will increase the likelihood that providers in any given area will accept new patients and be available to meet community needs [24]. Practices will need to expand availability to accommodate patients wanting to see their physicians after work. Embracing principles of the medical home (a primary care model that is patientcentered, comprehensive, teambased, coordinated, accessible, and focused on quality and safety) will allow patients and physicians to jointly determine the appropriate setting and timing of care [25]. Policymakers must also address the factors that influence Medicaid acceptance. Studies indicate higher Medicaid acceptance is associated with higher Medicaid reimbursement, lower Medicaid managed care penetration, and less perceived paperwork burden [26]. Our analysis suggests that Medicaid expansion without Medicaid reform may create a large insured population that may experience barriers to access.

Several limitations affect the internal and external validity of our study. As with any survey data, our findings are subject to recall bias, and these self-reported data do not provide means of validation. Furthermore, the MEPS does not allow us to comment on whether ED visits were necessary. Therefore, this analysis cannot determine whether having a USC or insurance type affects the appropriateness of emergency utilization. The survey participants are between the ages of 18 and 64 and earn less than 200% of the FPL, limiting the generalizability of our findings.

Further research is needed to determine why lacking a USC is associated with a greater reliance on EDs. If inadequate access to primary care is the primary mechanism, then we need to understand how workforce adequacy as well as state Medicaid policy, particularly around physician payment, may impact the likelihood of Medicaid recipients having a USC. Studies are also needed to explore the relationship between new delivery models (such as medical homes which promote comprehensive continuous relationships) and ED utilization. Geographic analyses are needed to identify variation in ED utilization and differential access to USCs, with qualitative explorations of causes. Finally, more studies are needed on the influence of insurance coverage and USC on the urgency of ED visits.

## 5. Conclusion

Those who lack USCs, particularly the uninsured and Medicaid enrollees, are more likely to rely on EDs. Health systems should support policies that expand access to USCs for low-income patients.

## Figures and Tables

**Table 1:** Number of emergency department (ED) visits, ambulatory care visits, and percentage with half or more of ambulatory visits to the ED, by insurance type and usual source of care (USC) (2000, 2011), unadjusted.

Year	Insurance type	Mean # of ED visits		Mean # of ambulatory visits		% of ambulatory care visits to the ED		% with half or more of ambulatory visits to the ED	
		USC	No USC	USC	No USC	USC	No USC	USC	No USC
2000	Private	0.22	0.37*	7.60	3.92*	5.8%	14.0%*	4.7%	12.8%*
	Medicaid	0.56	0.19*	9.53	7.00	10.9%	8.9%	9.5%	8.5%
	Uninsured	0.37	0.47	5.50	4.40*	12.4%	23.8%*	12.3%	25.5%*

2011	Private	0.28	0.30	6.99	4.39*	5.8%	8.6%*	5.0%	7.8%*
	Medicaid	0.61	0.65	10.01	5.04*	10.3%	21.8%*	8.1%	21.6%*
	Uninsured	0.43	0.51	6.15	3.75*	10.0%	23.4%*	8.8%	24.1%*

*P < 0.05 for difference between USC and no USC.
